# “The prayer circles in the air”: a qualitative study about traditional healer profiles and practice in Northern Norway

**DOI:** 10.1080/22423982.2018.1476638

**Published:** 2018-05-31

**Authors:** Anette Langås-Larsen, Anita Salamonsen, Agnete Egilsdatter Kristoffersen, Trine Stub

**Affiliations:** a The National Research Center in Complementary and Alternative Medicine (NAFKAM), Department of Municipality Medicine, Faculty of Health Sciences, UiT, the Arctic University of Norway, Tromsø, Norway; b Regional Centre for Child and Youth Mental Health and Child Welfare (RKBU North), The Faculty of Health Sciences, UiT the Arctic University of Norway, Tromsø, Norway

**Keywords:** Sami-healers, traditional healing, reading, blowing, curing, prayer, medical pluralism, health sectors, traditional knowledge

## Abstract

In Northern Norway, traditional healing has been preserved by passing down the knowledge through generations. Religious prayers of healing (*reading*) and Sami rituals (*curing*) are examples of methods that are used. We have examined traditional healers’ understanding of traditional healing, the healing process and their own practice, as well as what characteristics healers should have. Semi-structured individual interviews and focus group interviews were conducted among 15 traditional healers in two coastal Sami municipalities in Norway. The traditional healers understood traditional healing as the initiation of the patient’s self-healing power. This power was initiated through healing rituals and explained as the power of God and placebo effect. During the healing ritual, the doctor’s medical diagnoses, the patient’s personal data and a prayer in the name of The Father, The Son and The Holy Spirit were used in combination with steel and elements from the nature. The traditional healers stated that they had to be trustworthy, calm and mentally strong. Healers who claimed that they had supernatural abilities (clairvoyant or warm hands) were regarded as extra powerful. According to the participants in this study, the healers must be trustworthy, calm and mentally strong. Moreover, these traditional healers drew on information from conventional medicine when performing their rituals.

## Background

Traditional medicine (including traditional healing) may be understood as “the sum total of the knowledge, skills and practices based on the theories, beliefs and experiences indigenous to different cultures used in the maintenance of health, whether they are explicable or not, used in the prevention, diagnosis, improvement, or treatment of physical and mental illness” [1]. In Northern Norway, traditional medicine is influenced by Sami, Kven and Norwegian folk medicine, reflecting these populations understanding of nature, humanity and life [2]. It also mirrors a large part of the Sami cultural heritage through a belief in the universe and the origin of man [3]. Kristoffersen et al. [4] reported that 13.8% of the participants in a large population-based study in Northern Norway reported to have used traditional healing once or more during their lifetime. This number was considerably higher (25.7%) among the participants defining themselves as Sami. In Norway, traditional medicine is often understood as a kind of Complementary and Alternative Medicine (CAM), which is defined in Norwegian 1 Act No. 64 of 27 June 2003 relating to the alternative treatment of disease, illness, etc:

*Alternative treatment is understood as health-related treatment which is practised outside the established health services and which is not practised by authorised health personnel. However, treatment practised within the scope of the established health services or by authorised health personnel is also covered by the term alternative treatment when the methods used are essentially methods that are used outside the established health services* [5].

## The Sami culture

The Sami culture was closely connected to nature, and in this pre-Christian religion, the noaidi (traditional healer) played an important role. The noaidi was contacted and used as a counsellor, clairvoyant and healer. These healers were known for being able to handle both good and evil forces. Historically, the culture has been exposed to the authorities’ active Christian proselytisation. Their aim was to overcome the pre-Christian Sami nature religion. At the same time, the Sami and Kven culture were exposed to a comprehensive assimilation policy in which the school was the main focus. The aim of this policy was to abolish the Sami and Kven languages. In this process, the children were removed from their original environments and placed in residential schools where they were not allowed to speak Sami or Kven. One of the results was that many Sami and Kven became ashamed of their culture, and turned away from their language and culture. Over the past years, there has been an active revitalisation of the culture and language. This has helped increase the prestige of these cultures. This may also have provided greater open-mindedness regarding traditional healing [6].

The use of traditional healers has long traditions in the Sami population in Northern Norway [7–9]. One of the reasons for using traditional healers was that there were few medical doctors in Norway in the old days [10]. Even if the district doctor scheme was established in 1836 [11] the closest doctor could be more than 125 miles away [12]. Due to long distances and high costs, people seldom visited a medical doctor. Instead they used traditional healers who were locally rooted and provided their services free of charge [11]. The tradition of using traditional healers when becoming ill has been preserved up to the present [6,13–18].

The healers in Northern Norway are called *readers, blowers* or *curers* (guvhllàr in Sami). *Reading* is a ritual where the healer reads a prayer based on the Bible to relieve symptoms and complaints [15]. *Blowing* is a ritual where the healer blows three times after having read the healing prayer. Many healers claim that they are a tool for a godly power that works through them [15]. This practice is based on the Bible and the power of God. This power may be defined as God. However, sometimes this power is undefined [19]. According to the literature, they used inherited abilities (such as clairvoyance and warm hands) [14,15,19,20]. The Sami culture is diverse, and healing practices vary from one place to another. Therefore, there is still a lot we do not know about the healing tradition in Northern Norway.

The Sami generally have less academic education than the majority of the Norwegian population. This is especially true for the older generation. However, Sami and non-Sami people at the same level of education report few differences in sickness [21]. Therefore, there is no indication that the Sami and the majority of the Norwegian population differ much regarding health condition, prevalence of disease and life expectancy [22]. It seems like the difference in health between indigenous and non-indigenous people, that is quite common in other Western countries, is no problem in Norway [23]. According to Kvernmo, this can be attributed to the present socio-economic conditions of the Sami in Norway.

## What do we know about healers?

Before the christening of the Sami people, the Sami culture was associated with witchcraft and shamanism and was illegalised. Hætta interviewed three healers from a Sami area in South Troms [13]. She found that secrecy of the healing tradition had its roots in the witch process when the practitioners could be sentenced to death for conducting their work. Consequently, the rituals, rites and dissemination of the Sami tradition connected to healing were forced to silence and were only conducted privately [24]. The tradition preserved the christening of the Sami and was changed and preserved through Christianity as a Christian healing practice [6,14]. The practice was kept in silence till recent times [14]. There are, however, **degrees of secrecy**. Many Sami people can read verses and heal warts, and several have healers in their family that are able to read prayers to stop bleeding. Other healing rituals, however, are secret to most people [6]. Henriksen [18] interviewed healers from West Finnmark and Northern Troms. She found that the healing tradition was related to Christianity and not to the Sami. She expressed that *reading* was practical help for everyday life. Furthermore, she claimed that the healers should not boast of their own abilities according to biblical norms about boastfulness and abuse of the «sacred». In recent years, several studies among traditional healers in Northern Norway have been conducted [6,13,15,19,20]. In his study, Sexton [19] found that there was a big difference between the younger and older traditional healers. The younger healers more often combined traditional knowledge with modern healing when practising traditional medicine. (A few of the younger healers in the area held perspectives that were more global or composite. This also included obvious influences from sources, such as the Indian Chakra system, Native American Medicine or even modern knowledge of vitamins and minerals (understood as modern healing). Although these perspectives might be thought to be more alternative, they were held by people who were in a family line of healers and, also, practised healing as had been done by their parents or grandparents). Nergård [20] studied two traditional healers living in Finnmark for more than 10 years. They clamied that they have had special abilities, such as *warm hands* and *clairvoyance* since early childhood. Miller studied the transfer of knowledge between an older and a younger traditional healer in Porsanger in Finnmark [15]. The traditional healers in her study explained that healing takes place with the help from a higher power, and that they initiate the healing process within the patient. Myrvoll studied healing in a small Sami area in Nordland [6,14]. She stated that the healers’ abilities can be regarded as heritage or a gift of grace. When conducting the healing prayer, the healer often apply the Lord’s Prayer in combination with prophetic verses from the Bible. Various words are used for different diseases [25]. However, the exact wording is only shared between the apprentice and the master and within the healing municipality [19].

## Theoretical foundations

Communication is essential in consultations with patients as good communication is a prerequisite for making correct diagnoses. Kringlen and Finset [26] claim that patient consultations follow a certain three step pattern. The first step is (i) to build trust and map the patient´s problem. The second is (ii) to maintain the therapist–patient relationship. The third is (iii) to inform and initiate treatment. They connect (i) and (iii) to task-oriented aspects and (ii) to emotional aspects. Patient-oriented medicine is used to describe a communication style that ensures the maintenance of task-oriented, as well as emotional aspects during the consultation. This in contrast to an illness-oriented style, where the therapist is concerned with the disease as a purely biological phenomenon [27]. Kringlen and Finseth [26] argue, based on research, that patient-oriented medicine should maintain both task-oriented, as well as emotional aspects. This may be related to the difference between “healing” and “curing”. Healing refers to the whole person, or the whole body understood as an integrated system, including both physical and spiritual components. Curing, on the other hand, refers to an act of successfully treating a specific condition. Curing and healing may also be connected to the two concepts of “disease” and “illness”. Disease is concerned with the doctor´s understanding of disease, whereas illness includes the patient´s experience of the disease. Strathern and Steward [28] claim that biomedicine is primarily concerned with curing, whereas CAM modalities belong to the treatment philosophy of healing, in which some incorporate curing, and others do not. According to Helman [29], curing and healing are both included in practical clinical work. CAM therapists, as opposed to doctors, often spend more time with their patients [30]. This enables them to maintain the emotional aspects to a greater extent, which makes the patients feel like whole persons rather than purely biological phenomena. However, the essence of all CAM and conventional medical healing techniques lies in the ability to arouse the patients’ hope, increase their self-esteem, encourage the patients emotionally and strengthen their relationships to social groups. The knowledge of traditional healers from previous studies is based on data from few participants [13,14,17,19,20,31]. We therefore wanted to include a larger number of traditional healers in our study to examine possible diversities in the field. In this study, we will investigate healer profiles and healing practices seen from the perspective of the healer. What is traditional healing, how is it practised and which abilities are required for traditional healers? The study was conducted in the coastal areas of Northern Norway as sparse previous research from those areas exist.

## Methods

### Design

This is a qualitative study based on 15 individual interviews and one focus group interview among traditional healers in Northern Norway. Qualitative design is best suited to explore themes when limited previous knowledge exists [32]. Furthermore, a qualitative design is suitable when the aim is to examine the participants’ own understanding and experience of the phenomenon under investigation, which is traditional healing in this study [33]. Traditional healing is considered secret knowledge that is accessible only to a few people [13]. The first author of this study is Sami. Her previous research has shown that ethnic belonging and cultural knowledge are important to get access to traditional healers as study participants [13,16,34]. Researchers who are part of the culture are able to ask questions in a different way compared to researchers from other cultures. On the other hand, the researchers need to be focused and reflexive to be able to identify the natural and obvious in their own culture [35].

### Data analysis

Two of the participants did not want their interviews to be recorded. Instead, the first author took notes during these interviews. They were typed immediately after the interviews took place and forwarded to the participants for validation. Thirteen of the interviews were tape-recorded, and the first author performed the verbatim transcriptions [36]. The data was coded and analysed in NVivo 11 software [37,38]. The data was interpreted using a thematic content analysis. This method is best suited to analyse large amounts of text data [39]. According to Hsieh and Shannon [39], a content analysis is especially well suited to explore contrasts and similarities within the material. Initially, we used predefined codes (personal characteristics, training, what is healing? and tools). Other codes emerged throughout the research process (healing and conventional health care, the needs covered, advertising, diseases, faith, old traditions in the modern society). We identified stories, quotes and text segments that we linked to codes in NVivo [40]. These text condensations were extracted and inserted into a matrix according to themes that emerged from the material [38]. The first and last authors discussed and determined all steps in the analysis. The first author’s contacts translated the Sami words used by the participants into Norwegian. A professional translator translated the quotations into English.

### The participants

The participants (4 women and 11 men) were traditional healers from two Sami – Norwegian municipalities. The average age was 63.9 years. Three of the healers were somewhat younger than the others (43, 50 and 57 years old). These had a more modern view of traditional healing, and combined traditional healing and CAM. Two of the healers used Reiki healing as a supplement to traditional healing, and one of them used massage in his healer practice (Table 1). However, other young healers did not combine these modalities.

**Table 1.** Sample characteristics.

Participants	15 total
**Classification of ethnicity**	
Multi cultural group (Kven, Sami and Norwegian)	1
Sami 1 (speaking Sami or group affiliation)	12
Sami 2 (at least one parent or grandparent who speak Sami)	2
Norwegian ethnicity	0
**Gender**	
Female	4
Male	11
**Age** Female Male	**Average: 63.9 years** 64 years 70.3 years
**Grew up in the participating municipality**	
Grew up in the municipality	12
Grew up outside the municipality	3
**Profession**	
Manager	3
Academician	1
Office	2
Retired (academician, farmer, housewife)	7
Self-employed	2
**Language**	
Speak Sami	12
Sami language course	1
Parents who speak Sami as their domestic language	14
Grandparents who speak Sami as their domestic language	15
Kven	1
**Religious affiliation**	
Member of the State Church (Christian Protestants)	15
Affiliation to the Laestadian movement	12
**Healer training**	
Family of healers	15
Course	1
Spouse’s family	1
Combining traditional healing and CAM	3
Female	1
Male	2

The participants had grown up in the municipalities in which they presently lived, except three participants who had grown up in other parts of Norway where traditional healing is commonly practised.

### Ethnicity

There have always been people of different ethnic backgrounds in Northern Norway [41]. They speak different languages and/or belong to different cultures, such as Sami, Kven (Finnish descent) and Norwegians. In this study, we chose to divide the participants into three categories in line with other Norwegian researchers [42]:

Group 1: *Sami 1*: Those who defined themselves as Sami by speaking the Sami language, felt connected to the Sami by speaking their language or felt affiliated with the Sami.

Group 2: *Sami 2*: Those who had at least one parent or grandparent who speak Sami, or felt a personal Sami connection (language or a feeling of affiliation).

Group 3: *Mixed ethnicity/multi-cultural group*: Those who had mixed ethnicity who could tell that they had at least one grandparent who spoke Kven or Sami, had a minimum of one Sami and one Kven parent or grandparent, or expressed a sense of affiliation (language or sense of belonging).

In our material, 12 participants were defined as Sami 1, two as Sami 2 and one as mixed (Sami, Norwegian and Kven). They all came from a family line of traditional healers. Three of the participants had senior positions in the municipalities. One held an academic position, two worked as office clerks and two were self-employed. Seven of the participants had retired (former farmers, academicians and housewives). They were all members of the State church (Christian Protestants), and 12 of them had affiliation to the Laestadian movement (Table 1).

### Recruitment

Prior to this study, the research team had an introductory meeting with the managers of the municipalities. The municipality health-care managers had a positive attitude towards the study and informed potential participants about it. Public meetings were held in the municipalities, and information about the study was published on the local radio station and in the local newspapers. This approach is in line with Porsanger and Guttorm [43] who emphasised the importance of local roots and cooperation when researching in the Sami areas.

The recruitment took place by snowball sampling [44], which means that a person knows some people who recommend participation to others. This method of recruitment is well suited to explore confidential themes in limited-entry environments [33]. Most of the previous research on traditional healers has included one to eight healers [13–15,17,20]. In this study, a total of 15 healers of both genders formed the basis for the qualitative analyses. We wanted to examine possible diversities in the field, and the aim was to include a larger number of traditional healers [45]. However, after 15 interviews, we did not receive any new information about the topic of interest. We therefore concluded that we had achieved saturation, and no more healers were included in the study [46].

### The interview guide

The interview guide contained the following themes: *The healer’s assignment; Experience with patient help seeking; What kinds of illness do they seek help for; Understanding of illness and healing; Healing practice; share and transfer knowledge; Ethnicity.*

### Individual interviews

The first author conducted 15 individual interviews to find out about the participants’ experience with, and understanding of traditional healing [44]. Five of the interviews were conducted in the participants’ workplace and 10 were conducted in the participants’ home. In four of the interviews, the participants’ spouses were present. The average interview lasted an hour, but the visits could last for several hours (including food and beverages). Two of the participants were interviewed several times as they wanted to elaborate on the information they had previously shared with the researcher. These interviews lasted between 2 and 4 h. The researcher asked questions from the interview guide while she gave the participants the room to elaborate on issues of their interest. All participants were given the opportunity to read and comment on the transcribed material from their own interview.

### Focus group interview

A focus group interview, based on the same interview guide, was conducted with a family of healers who had not been previously interviewed. The criteria to choose this family were that they were easily available and that they had knowledge about how the healing practice had developed over time. The family consisted of five people: a married couple, one daughter, a grandmother and an uncle. Their ages ranged from 30 to 70. The interview was conducted in their home. Two of the participants were healers. According to Steward and colleagues [47], family safety may make it easier for the participants to share complete information on cultural and sensitive themes. Sensitive themes are often shared naturally between the generations of the family [34]. In addition, Malterud [32] stated that when conducting group interviews, the researcher gets access to more abundant and distinct information. Traditional healing is a sensitive and secret tradition. Therefore, the first author conducted the focus group interview without including a moderator [48].

### Interview guide

A semi-structured interview guide was used as a template during the interviews, as it allows deep and more personal replies [36]. Semi-structured interviews are well suited to gather information about the participants’ lifeworld. Such an approach also makes it possible to linger or elaborate on issues raised by the participants [36]. The interviews were conducted as natural conversations, while the researcher made sure that all the questions in the interview guide were answered.

Six themes were identified: *Personal qualities and characteristics, healing practice* and *the use of tools, explanation models, natural talents* and *heritage*. Three of the themes (*heritage, personal characteristics* and *explanation models*) were predefined. The other three (*natural talents, clinical practice* and *the use of tools)* emerged from the analysis process.

### Ethics

The study was approved by Norsk Senter for Forskningsdata (the Norwegian Centre for Research Data) project number 38334. This study meets the standard of the Helsinki Declaration of 1975, last revised in 2013 [49]. Sami and Kven have been exposed to a brutal assimilation policy, and a former research used to classify and emphasise ethnic hierarchy and dominance. Even though the policy of Norwegianisation and the offensive research conducted on ethnic minorities in Norway is over, the consequences of this policy are still stuck in people’s minds. This has contributed to a collective pain in the Sami and Kven populations. This demands particular sensitivity when conducting research among these populations [7]. To receive tribal approvals, two members of the research team visited both municipalities. There they arranged an information and a coordination meeting with the managers and health care managers of the municipalities. Local resource persons and cultural bearers in the municipality were consulted. The participants were thoroughly informed of the purpose of the study, which was to focus on the particular and health promoting aspects of using traditional healing. To protect the participants and sustain anonymity, the participants were given fictitious names. (They were given the opportunity to have real names, but they refused.) They were also given the opportunity to read and approve of their own interviews. They have all given their consent to publish. Written informed consent was obtained from all participants.

## Results

We will present the results distinguishing between healer profiles and healing practice.

### Personal qualities and characteristics

According to the participants, traditional healers must be trustworthy so that people can gain confidence in them. They needed to keep calm even in difficult situations. Therefore, it is important that the traditional healers are *confident* and *calm*. In addition, they must act with authority when people experience crises and need support. Sigurd explained:

If you experience a strong wind one day, then you know it’s blowing today, but tomorrow it will be calm. That means that you must know inside yourself that it will pass. If you don’t understand that, then you’ll make mistakes and hasty things. Yes, all the time you need to know that things will change for the better. So you need to be able to cope with the strong wind when it’s blowing (Sigurd).

They need the ability to show *sympathy* and *compassion* and put themselves in the place of the ill people. Lena used a metaphor to explain this:

If a child’s in pain, the mother assimilates the pain. The child can see in the mother’s eye that her mother hurts because of her pain. Then the mother lets go of the pain, and then the child can let go too. In this way the mother regulates the child emotionally, and the child no longer feels alone with her pain (Lena).

The same happens when a healer *reads* for a person:

“[…] you visit a ‘reader’ with your worries, and the pain is taken seriously, both your own fear, pain, and all. And good ‘readers’ assimilate all of this. Then you don’t have to carry it alone (Lena)”.

Empathy and the ability to see another person are good qualities for traditional healers. To handle difficult situations safely, they had to take control and exert authority.

### Natural talent

Several of the participants claimed that they had special healing abilities, such as *warm hands*. These abilities were discovered during their childhood. Step by step the elder healers taught them healing verses.

*No, I had no words, I didn’t have any words, I only had warm hands (Anna).*

Nils elaborated:

I actually feel it when I lay my hands on a person, then I feel it in my hands that the person’s strongly responsive to what’s going to happen. Then I feel that my hands simply start glowing. Then the channel’s wide open which enables me to transmit impulses that might have healing effects (Nils).

In the Sami culture, special abilities were connected to clairvoyance. If you had such abilities, you could look into the future, get warnings of deaths or tragic happenings, and also be able to interpret dreams. The healers who have such abilities are considered extra powerful and have a high status.

*And if you had dreams that foretold the future, then you were considered special (Marit).*

### Heritage

The traditional healers in our study come from different families of healers. Most of the participants have healers in their family, such as a father, a mother or a grandparent. The healers told of many people who had travelled a long way and lined up to visit their father or mother who were traditional healers, or that mum or dad received many phone calls from people who wanted help.

“It’s been passed down from generation to generation, from my grandfather, my father, and now me. We were eleven brothers and sisters, and I’m the only one who got those abilities (Anna)”.

“I remember that people kept coming all the time, and I often talked with them while they were waiting, drinking coffee. I often served them coffee (Knut)”.

To prevent the knowledge of healing from vanishing, it is important to pass this knowledge on to the next generation. Therefore, many healers in our study chose to pass this tradition on to their children. This often took place when they grew old or became ill, and when they realised that one of the children had such abilities. However, the younger generation did not always want to receive this knowledge:

“Yes, I’ve taught some of it to one of my children. When I became ill, I thought it was important to pass it on (Knut)”.

”My daughter has those abilities. I’ve told her, but she’s not interested. She couldn’t care less. She doesn’t even want to talk about it (Håvard)”.

## Healing practice and the use of tools

According to the participants in our study, nothing limits the use of *reading*. It is used to remove warts, rashes, neck and back pain, headaches, cancer and heart diseases. *Reading* is practised by reading a specific prayer to heal a person. The healing verses are kept secret to anyone except the healers. “The exact wording of the healing verses is not commonly shared”.

We use the word of God, that’s what we do. We use Bible texts, and the word of God, The Lord’s Prayer, and the Trinity. These verses do not belong to everybody. This is a kind of secret that I must preserve (Martin).

When the healers *read* a healing verse, they use the patient’s Christian name, date of birth and *diagnosis*. If the patient is about to undergo surgery, *the date for the surgery* will be stated. In addition, some healers use the patient’s address and the name of the hospital to which the patient is admitted. One of the participants explained:

No, I have my own way of ”reading”. Then I need the person’s date of birth, and I use my own words, words that I don’t tell anyone, and that I use as a tool to improve the present situation (Nils).

“Family names aren’t used when you’re baptized. So I don’t use family names at all when I read (John)”.

*You need the patient’s full name, age, and address (Martin).*

Various healing verses are used depending on what is to be healed. Some verses are used for blood stopping, others for pain relief and removal of inflammation. The Lord’s Prayer is often used in addition to making the sign of the cross while praying in the name of the Father, Son and The Holy Spirit. Anna explained:

“Well, you know, you just lay your hand on the patient and pray the prayer you’ve been taught, and it works. They (the Sami people, authors comment) had great faith in words, different words …. (Anna)”.

Interviewer: *The word of God?*

*Yes. Quite right. They believed very much in that (Anna).*

People, who are seriously ill, such as cancer patients, often contact healers. People want help to heal the cancer itself, but also help to lessen the adverse effects of the cancer treatment, such as nausea, pains and anxiety. The healers then include the *cancer diagnosis* and *possible new test results* in their practice. Peder uses steel (knife) prior to and after surgery. He told us:

*However, in cases of surgery you have to use a knife (Peder).*

Another explained:

*You’ll be protected and you take away the bad energy with the knife (Marit).*

The term *cure (Norwegian: kurering)* is often used in the Sami healing tradition. This term is used when the traditional healers practise *reading*, in addition to using elements, such as *earth, fire* or *water* in their healing rituals. This means that they seek help from, and use materials from nature when they cure. Examples of things that are used are *moss* (which needs to be collected from a swampland where you cannot see the sea), *earth, water* and *stones*. In addition, organic materials, such as *wool* and *potatoes* are used.

The patient needs to bring a piece of woolen yarn and some earth in a bag. Then I touch each wart with some of the woolen yarn. Then I tie a knot in the woolen yarn, and I grab the pieces of earth and put them on the wart. The rest of the earth and the woolen yarn must be packed in a bag and brought back to the place where they were found. When the woolen yarn is well disintegrated, the warts will also be gone (Nils).

When conducting *cure*, old Sami traditions are used combined with elements from Christianity, such as prayers and making the sign of the cross.

*You need steel and God’s words (Martin).*

Interviewer: *Steel and God’s words?*

“Yes, because all evil is afraid of steel. Then you have to say that you’ll take away all evil, that is from the wound (Martin)”.

The traditional healers pass the knife facing the injured area. This is repeated three times. They say a prayer to God about healing while they ask the steel to remove the pain from the patient. Some traditional healers also use steel as a means to cure tendonitis and pulled ligaments. One of the healers explained:

*You have to use the knife, and then you make the sign of the cross (Martin).*

If the patients needed internal cure, the healers often used *water*. (Internal cure is needed when patients suffer from internal diseases, such as cancer, diabetes or chronic fatigue syndrome.) The healers read a prayer over the water and gave it to the patients in a bottle. Then the patients drank the water three times a day. The idea is that the water works as if the healers read a prayer for the patients several times a day. Anders and Anna elaborated:

*Yes, some read to cure internal illness. Then you can read in water (Anders).*

“Once a man came from Bodø, along with his son. He (the son, authors comment) had chronic fatigue syndrome. Then I read in water. I can read in water so that you get it inside your body (Anna)”.

*Earth* is used as a means to cure. The principle is that if the patient has rashes from having been in contact with earth, the healers use earth to remove the rashes. Then earth is used as a supplement to healing prayers. As with *earth, stone* is used if the patient has been injured by a stone. Then the traditional healers ask the stone to remove the pain it has caused. They explained:

Yes, I learnt that too from my dad, that I could use steel and stone and earth. If you had a rash because you’d been in contact with earth, then you could use earth. If you’d been injured by a stone, then you could use a stone to remove the pain (Anna).

“Rashes mostly come from being in contact with earth. Then you can use some of the earth, grass, or something like that to cure (Peder)”.

Stone may also be used if the patients have been infected by polluted water. The stone that is used must come from the same polluted water. The healers pass the stone around the infected area while they recite a healing verse and make the sign of the cross using the stone. This is called «*gierret»* in the Sami language. One of the healers explained:

The idea is that you collect stones from the water, preferably three stones. Then you perform «gierret» using the stones. You ask the stones to remove the pain. Then you return to the location where you found the stones and throw them back in. It’s important that you put them in the exact same place as you found them (Martin).

*Cupping* and *bloodletting* were previously used, but are no longer used. Martin explained about this tradition:

[….], but bloodletting using a spring-loaded lancet was as a matter of fact, commonly used in the Norwegian culture. Some believed you had to withdraw the nasty, black blood. […] Withdraw a little bit. Some were especially good at this. They knew which blood vein on the leg to use. The right leg would do something for earaches, and so on (Martin).

Traditional healing uses elements from old Sami traditions and Christianity. In clinical practice, elements, such as earth, stone and water are used. The healing rituals include steel and the word of God. Both elements play important roles.

## Explanation models

The participants in our material were of the opinion that healing takes place through *the power of prayer* and *God’s Word* in the Bible. The healers are tools for the divine power running through them. They explained:

*[…] if someone gets well/cured, God is to be honored. We are merely mediators (Andreas).*

*God Almighty can heal and the prayer circles in the air – south and north and east and west (Inga).*

It is important that the patients *believe* that the healers can heal. However, it is equally important that the healers believe in what they are doing, that they believe in God’s Word. Martin explained that there are three elements present in cure:

So we’ll be three persons present, the patient, the “reader”, and what happens. It’s the cure that’s the third person. This means that the cure is an existence. The patient and the healer share a common faith in a successful cure (Martin).

The traditional healers stated that *reading* and *cure* provide patient safety, and the reader is a person with whom they can share their problems. Martin expressed that *reading* mobilises the patients’ own power, and that there is much healing power in the expression *I want to become healthy!*

John also referred to the *placebo effect:*

“[…], so there’s a little bit of placebo as well. It may also be helpful for many people just to know that they have someone to contact (John“)”.

Prayer and faith in God’s healing power are the corner stones of the North Norwegian *reading* tradition. In addition, the healers need to believe in what they do. Another important element in that healing tradition is to stimulate the patients’ self-healing power.

## Closing remark

We found few differences regarding the age of the healers in the two municipalities. However, we interviewed more female healers in one of the municipalities. We found differences in the healing with other methods that were used. Three of the healers in one of the municipalities combined traditional healing with other CAM modalities. We do not believe that this is subject to different traditions in the two municipalities, but rather the fact that these healers were younger and therefore more open-minded towards modern CAM modalities.

## Discussion

This study gives insight into how traditional healers in two coastal Sami municipalities understand traditional healing and their opinion on typical characteristics of traditional healers. The participants emphasised the importance of being trustworthy, mentally strong and exerting authority when conducting *reading*. According to the participants in this study, the patients first visited a doctor to get a diagnosis when they became ill. Then they went to see a traditional healer to have him or her *read* against the illness. Furthermore, the healer used the diagnosis and possible test results when performing *reading*. These are new and interesting findings not known before, showing that these traditional healers drew on information from conventional medicine when performing their rituals. Other Norwegian studies confirm that the patients seek help from traditional healers as well as from doctors when they become ill [15,18,31]. They also reveal that the healers are careful not to compete with biomedicine [18]. Several international studies show that patients use different healing traditions alongside with biomedicine [50–58]. However, the inclusion of biomedical diagnoses and test results in the healing ritual is new information based on this study.

People often seek help from CAM therapists because they have already built trust and established a good therapist–patient relationship. The focus in modern medicine, however, has been more on technology. The traditional healers in our study understood healing as the initiation of the patient’s self-healing power. According to Strathern and Steward, healing is as an intrinsic (activation of the patient’s self-healing power), as well as an external process (activation of the power of God). The third part is the cure, which is the powerful and active impulse in the healing process. The healer is only the mediator of this impulse [28]. These findings are in line with other Norwegian research of healing [31]. The aim of a recent study was to gain knowledge about how CAM providers understand the placebo effect and its position in the healing process. This study found that the patients’ positive beliefs and expectations about the treatment play a significant role in the self-healing process. The CAM providers understood placebo effect as the patient’s self-healing power, resulting from establishing trust and belief in the treatment process [30]. Moreover, the therapist–patient relationship becomes more equitable if both parties respect one another. It is often easier to feel respect for the patients when they belong to the same culture and social group as the therapist [26].

In addition, Nergård [17] found in his material that healers interpreted healing as a Christian phenomenon, but also as an ancient Sami phenomenon where guardian angels act as helpers in the healing process.

As mentioned earlier, traditional healing is not a practice open to anyone. Until today it has been preserved through secrecy. Our findings show that the present traditional healing takes place within a Christian context. We examined traditional healing in areas where Laestadianism [59] has played and still plays an important role. The Sami became Christians through the *Laestadianism*. Laestadianism is a Christian layman’s movement that is named after the Swedish priest Lars Levi Laestadius (1800–1861). Laestadius was of Sami descent and had thorough knowledge of the Sami language, culture and way of living [59]. The Laestadian revival was rapidly spread among the Sami people and throughout large parts of the Northern hemisphere by the Sami reindeer herders [60]. The Sami language and culture have been preserved through this movement [59–61]. Fifteen healers professed themselves Christians (12 of them were related to Laestadianism). This is in line with findings from other research [13–15,17,31,62]. This movement may have shaped their methods, views and interpretations.

### The healing process

The healers in our study used secret healing prayers, including the person’s name, birth date, diagnosis, time for surgery and address when they *read* against illness. Dependent on the severity, they combined the Christian rituals (The Lord’s Prayer and the sign of the Cross) with Sami knowledge (*curing)* in which natural elements such as steel, water, moss, earth and stones were used. This is in line with researchers [7,19,31,62] who describe a ritual in which the healer read to moss and thereby passed it across the eczema on the patient’s body. Mathisen [2] and Nymo [31] describe a ritual in which the healer used three stones from different places in the nature (from the hill, the cemetery and the shoreline). When the ritual was completed, the stones were replaced exactly where they were found. The explanation for this was that whatever has been borrowed from the nature must be returned back to the nature. Mathiesen explained that when the stone is returned back to the element that caused the illness, both the element and the spirit of the illness would take the illness back [56]. Henriksen explained that the steel is a symbol of force, strength and power, and that the knife is an instrument that the healer uses to enable God to heal the person [23].

### Training

Elderly relatives often pass on training when they discover a person in possession of abilities beyond what is normal or who is especially open towards a spiritual dimension [14,15,17,19]. Previously, it has been demonstrated that a decisive factor for passing on this knowledge to another person was that he or she showed signs of, interest for and respect for the tradition, and was able to keep this knowledge secret [13]. This is in line with other researchers who found that the healers were carefully chosen based on personal characteristics and abilities [18,31]. The rituals and healing verses are taught when the environment finds out that a person has healing abilities [13,15,20]. These are not open to anyone, but today there is more openness about this tradition [6].

### Characteristics

According to the healers in our study, traditional healers should be mentally strong and trustworthy to be able to keep the healing verses and confidential information secret. According to Hætta [13], it is important that the healers have high moral standards as they have to deal with good as well as bad power. In line with our findings, Hætta [13] expresses that healers must have an inner desire to help others and also be spiritually oriented.

## Implications for practice

There is sparse research about Sami healers, and there might be big cultural variations and expressions within the Sami culture. To gain increased knowledge of the similarities and differences, future studies should include healers from several Sami areas and across the national borders within Sapmi (the land of the Sami across Norway, Sweden, Finland and Russia). They should also include questionnaires combined with qualitative interviews of healers and users of traditional medicine in the entire Sami area.

### Strengths and weaknesses of the study

The study was supported by the municipality management of both municipalities. The study is a part of a larger research project that includes health personnel, healers and patients in these two municipalities. It has been essential for us to obtain acceptance and cooperation with the municipality health care managers and Sami key persons as local roots is especially important when studying indigenous people [43,63,64]. The managers of the municipalities have contributed with counselling and offices for public meetings. They have also drawn attention to the study through information on websites and other local arenas, such as the library, communal notice boards and local media. The first author is Sami and has her roots in one of the municipalities. Therefore, she knows the Sami culture and traditional healing. Studies have shown that the researcher’s Sami cultural background is of importance when it comes to trust and entry into the research field [13,19,31]. On the other hand, this might also be a weakness. As a researcher of your own culture, it may be difficult to get hold of the obvious in that culture [35]. Still we think that the total experiences of the authors contribute to analytical depth and distance. The second author has her roots in the other municipality where the study took place. She has a background in sociology. The third author is a healer, and the last author is a CAM provider (acupuncturist and homeopath).

## Conclusion

According to the participants in this study, the healers must be trustworthy, calm and mentally strong, as well as open to a possible spiritual dimension. The healers functioned as tools or channels for the power of God. This power was initiated through healing rituals and explained as the power of God and a placebo effect. Depending on the disease and severity, Christian healing prayers were combined with Sami healing rituals in which doctor’s diagnoses and test results were actively used.

## References

[1] World Health Organization (WHO) Traditional medicine: definitions Geneva: World Health Organization; 2014 [cited 2017 817] Available from: http://www.who.int/medicines/areas/traditional/definitions/en/.

[2] Mathisen SR. Folkemedisinen i Nord-Norge: kulturelt felleskap og etniske skiller. English: the folk medicine in Northern Norway: cultural community and ethnic divisions In: Altern I, Minde GT, editors. Samisk folkemedisin i dagens Norge: rapport fra seminar i regi av Institutt for sosiologi og Senter for samiske studier. Vol. 9 Tromsø: Senter for samiske studier; 2000 p. 15–13.

[3] nifab.no. Samisk folkemedisin. English: Sami folk medicine nifab.no: nifab.no. 2013 [cited 2017 628]. Available from: http://nifab.no/behandlingsformer/samisk_folkemedisin/bakgrunn.

[4] Kristoffersen AE, Stub T, Melhus M, et al Prevalence and associations for use of a traditional medicine provider in the SAMINOR 1 Survey: A population-based study on health and living conditions in regions with Sami and Norwegian Populations. BMC Complement Altern Med. 2017;17(1):530. 29233186 10.1186/s12906-017-2037-0 PMC5728044

[5] Act No. 64 of 27 June 2003. Act relating to the alternative treatment of disease, illness etc, Ministry of Health and Care Services, 2003-2003 Sess. 2003.

[6] Myrvoll M Traditional Sámi healing heritage and gifts of grace In: Miller BH, editor. Idioms of Sámi health and healing. Edmonton: Polynya Press; 2015 p. 47–69.

[7] Turi J Mui’talus sámiid birra: English: johan Turi - an account of the Sámi. Stockholm: Skolöverstyrelsen; 1965.

[8] Holmberg U Lapparnas religion (Lappalaisten uskonto, 1915). English: the Sami religion. Uppsala: Uppsala University, Centre for Multiethnic Research; 1987.

[9] Kvandahl H, Jonassen M, Thomassen N, et al Samenes historie: 2. English: the Sami History: 2. Trondhjem: Forfatterne; 1932.

[10] Norges offentlige utredninger. English: Official Norwegian Paper. Alternativ medisin. English: Alternative medicine. NOU 1998:21.Utredning fra et utvalg oppnevnt av Sosial og helsedepartementet april 1997. English: Report made by a committee appointed by Social and Health department April 1997. Avgitt til Sosial og Helsedepartementet 15. desember 1998. English: Delivered to Social and Health Department December 1998. Oslo. Statens forvaltningstjeneste. English: Government administration; 1998

[11] Sandvik H The district practitioner (1836-1984). Tidsskrift for Den Norske Laegeforening: Tidsskrift for Praktisk Medicin, Ny Raekke. 2000;120(26):3160–3161. 11109364

[12] Holm O Fra en nordlandsk prestegaard: oplevelser og skisser. English: from a North Country vicarage: experiences and anecdotes. Kristiania: Aschehoug; 1923.

[13] Hætta AK Secrecy in Sámi traditional healing In: Miller BH, editor. Idioms of Sámi health and healing. Edmonton: Polynya Press; 2015 p. 25–45.

[14] Myrvoll M. Bare gudsordet duger. Om kontinuitet og brudd i samisk virkelighetsforståelse. English: Only the word of god suffices. Continuity and change in Sámi world view. Tromsø: Universitetet i Tromsø; 2010.

[15] Miller BH Connecting and correcting: a case study of sami healers in porsanger. Leiden: University of Leiden; 2007.

[16] Larsen AL, Foss N Helsepersonell og “læsing”: hva forstår de, hva får de vite og hvordan forholder de seg til det? English: conventional healthcare providers and “reading”: what do they understand, what do they get to know and how do they relate to it? Luotta: sáme oahppása ja dålusj bátsadisá; 2013 p. 99–106.

[17] Nergård JI, Eriksen J Den levende erfaring: en studie i samisk kunnskapstradisjon. English: the lived experience: A study of Sami knowledge tradition. Oslo: Cappelen akademisk; 2006 p. 269.

[18] Henriksen AM, Brun A Å stoppe blod: fortellinger om læsing, helbredelse, varsler og hjelpere. English: stopping blood: narratives about reading, healing, foreseeing and helpers. Oslo: Cappelen Damm; 2010 p. 285.

[19] Sexton R, Stabbursvik EAB Healing in the Sami North. Cult Med Psychiatry. 2010;34(4):571–589. 20862528 10.1007/s11013-010-9191-x PMC2952111

[20] Nergård JI Det skjulte Nord-Norge. English: the secret Northern Norway. Oslo: Cappelen Akademisk Forlag; 2005 p. 150.

[21] Lund E, Melhus M, Hansen KL, et al Population based study of health and living conditions in areas with both Sámi and Norwegian populations–the SAMINOR study. Int J Circumpolar Health. 2007;66(2):113–128. 17515251 10.3402/ijch.v66i2.18241

[22] Hansen BB, Hamran T Helse- og omsorgstjenester til samiske eldre: temahefte. English: health and care services for Sami Elders: pamphlet. Tønsberg: Aldring og helse/Senter for omsorgsforskning Nord, UiT Norges arktiske universitet; 2015.

[23] Kvernmo S Tiden er et skip som ikke kaster anker”; Utvikling av helse- og sykdomsbildet og helsetjenester i den samiske befolkningen. English: “Time is a ship which doesn’t let go of the anchor”. Development of overview of health and illness and health services among the Sami population. Utposten. 2014;43(6):39–43.

[24] Hansen LI, Olsen B Samenes historie fram til 1750. English: the Sami history until 1970. Oslo: Cappelens Forlag AS; 2005 p. 672–8 p.

[25] Mathisen SR Folkemedisin og folkelige behandlere i det nordlige Norge. English: folk medicine and popular therapists in Northern Norway. Bergen: Etno-folkloristisk institutt, Universitetet i Bergen; 1986.

[26] Kringlen E, Finset A Den kliniske samtalen: kommunikasjon og pasientbehandling. English: the clinical conversation-communication and patient treatment. 2nd ed. Oslo: Gyldendal akademisk; 2006.

[27] Stewart MA Effective physician-patient communication and health outcomed: a review. Can Med Assoc. 1995;152:1423–1433. PMC1337906 7728691

[28] Strathern A, Stewart PJ Curing and healing: medical anthropology in global perspective. 2nd ed. Durham North Carolina: Carolina Academic Press; 2010.

[29] Helman CG Placebos and Nocebos: the cultural construction of belief In: Peters D, editor. Understanding the placebo effect in alternative medicine Theory. practice and research London: Churchill Livingstone; 2001.

[30] Stub T, Nina F, Liodden I Placebo effect is probably what we refer to as patient healing power. A qualitative pilot study among complementary therapists in Norway. BMC Complementary Altern Med. 2017;12(1):262. 10.1186/s12906-017-1770-8 PMC5429571 28499371

[31] Nymo R Helseomsorgssystemer i samiske markebygder i nordre Nordland og Sør-Troms: praksiser i hverdagslivet: “En ska ikkje gje sæ over og en ska ta tida til hjelp”. English: health-care Systems in Sámi woodland parishes of northern Nordland County and southern Troms County: everyday life practices: “You should not succumb and you should give yourself time. Tromsø: Universitetet i Tromsø, Det helsevitenskapelige fakultet, Institutt for helse- og omsorgsfag; 2011.

[32] Malterud K Qualitative research: standards, challenges, and guidelines. The Lancet. 2001;358(9280):483–488. 10.1016/S0140-6736(01)05627-6 11513933

[33] Malterud K Fokusgrupper som forskningsmetode for medisin og helsefag. English: focus groups as research method for medicine and health sciences. Oslo: Universitetsforlaget; 2012 p. 164.

[34] Larsen AL Fortellinger om læsing. English: stories about healing (reading). Tromsø: Universitetet i Tromsø; 2012.

[35] Paulgaard G Feltarbeid i egen kultur: innenfra, utenfra eller begge deler? English: fieldwork in own culture: from within, externally or both? In: Wadel C, Fuglestad OL, Aase TH, et al, editors. Metodisk feltarbeid: produksjon og tolkning av kvalitative data. Oslo: Universitetsforl; 1997 p. 70–93.

[36] Kvale S, Brinkmann S, Anderssen TM, et al Det kvalitative forskningsintervju. English: the qualitative resarch interview. Oslo: Gyldendal akademisk; 2009.

[37] Richards L Handling qualitative data: a practical guide. 3rd ed. London: SAGE Publications Ltd; 2015.

[38] Creswell JW Qualitative inquiry & research design: choosing among five approaches. 3rd ed. Los Angeles: Sage; 2013.

[39] Hsieh HF, Shannon SE Three approaches to qualitative content analysis. Qual Health Res. 2005;15(9):1277–1287. 16204405 10.1177/1049732305276687

[40] Malterud K Systematic text condensation: A strategy for qualitative analysis. Scand J Public Health. 2012;358:795–805. 10.1177/1403494812465030 23221918

[41] Det kongelige kommunal- og regionaldepartementet. English:Ministry of Local Government and Modernisation. Om samepolitikken. St. meld.nr.55. English: about the Sami policy In: Det kongelige kommunal- og regionaldepartementet. English: Ministry of Local Government and Modernisation, editor. Oslo: Det kongelige kommunal- og regionaldepartementet; 2000 1–209.

[42] Bakken K, Melhus M, Lund E Use of hypnotics in Sámi and non-Sámi populations in Northern Norway. Int J Circumpolar Health. 2006;65(3):261–270. 16871832 10.3402/ijch.v65i3.18098

[43] Guttorm G, Porsanger J Working with traditional knowledge: communities, institutions, information systems, law and ethics: writings from the Árbediehtu pilot project on documentation and protection of Sami traditional knowledge. Guovdageaidnu: Sámi allaskuvla; 2011.

[44] Polit DF, Beck CT Nursing research: generating and assessing evidence for nursing practice. London: Wolters Kluwer/Lippincott Williams & Wilkins; 2008 p. 796.

[45] Malterud K Kvalitative metoder i medisinsk forskning: en innføring. English: qualitative methods in medical research: an introduction. 3rd ed. Oslo: Universitetsforlaget; 2013 p. 238.

[46] Malterud K, Siersma VD, Guassora AD Sample size in qualitative interview studies. Qual Health Res. 2016;26(13):1753–1760. 10.1177/1049732315617444 26613970

[47] Steward DW, Shamdasani PN Focus groups theory and practice. London: Sage Publications; 1990.

[48] Morgan DL, Krueger RA The focus group kit. London: SAGE Publications Ltd; 1998.

[49] The World Medical Association Declaration of Helsinki - Ethical principles for medical research involving human subjects1964 revised 2013 24.05.1624. 05.16].Available from: http://www.wma.net/en/30publications/10policies/b3/.

[50] Kiesser M, McFadden J, Belliard JC An interdisciplinary view of medical pluralism among Mexican-Americans. J Interprof Care. 2006;20(3):223–234. 16777790 10.1080/13561820600718055

[51] Kaptchuk TJ, David M, Eisenberg DM Varieties of healing. 1: medical pluralism in the USA. Ann Intern Med. 2001;135(3):189. 11487486 10.7326/0003-4819-135-3-200108070-00011

[52] Marian F Medical Pluralism: global perspectives on equity issues. Forschende Komplementärmedizin/Research Complement Med. 2007;14(S02):10–18. 10.1159/000112319 18219205

[53] Albert S, Porter J Is ‘mainstreaming AYUSH’ the right policy for Meghalaya, northeast India? BMC Complement Altern Med. 2015;15:288. 26283420 10.1186/s12906-015-0818-x PMC4539927

[54] Lambert H Medical pluralism and medical marginality: bone doctors and the selective legitimation of therapeutic expertise in India. Soc Sci Med. 2012;74(7):1029–1036. 22336733 10.1016/j.socscimed.2011.12.024

[55] Elder WG, Crooks DL, Matheny SC, et al Toward interdisciplinary care: bridging the divide between biomedical and alternative health care providers. Ann Behavioral Sci Medical Edu J Assoc Behav Sci Med Educ. 2008;14(2):56–61. PMC4550219 26321860

[56] McGrath BB Swimming from island to island: healing practice in Tonga. Med Anthropol Q. 1999;13(4):483–505. 10626277 10.1525/maq.1999.13.4.483

[57] Teuton J, Dowrick C, Bentall RP How healers manage the pluralistic healing context: the perspective of indigenous, religious and allopathic healers in relation to psychosis in Uganda. Soc Sci Med. 2007;65(6):1260–1273. 17521791 10.1016/j.socscimed.2007.03.055

[58] Lieban R Urban philippine healers and their contrasting clienteles. Int J Comp Cross-Cultural Res. 1981;5(3):217–231. 10.1007/BF00050769 7318486

[59] Kristiansen RE Samisk religion og læstadianisme. English: sami religion and Laestadianism. Bergen: Fagbokforlaget; 2005.

[60] Andersen KT Den læstadianske vekkelsen i Tysfjord fra 1850 til andre verdenskrig. English: the Laestadian movement in Tysfjord from 1850 to World War II. Drag: Báhko; 2007.

[61] Evjen B, Hansen LI Nordlands kulturelle mangfold: etniske relasjoner i historisk perspektiv. English: nordlands cultural diversity: ethnic relations in historical perspective. Oslo: Pax; 2008 p. 348.

[62] Sande H, Winterfeldt S Four Sami healers: a preliminary interview study. Nord J Psychiatry. 1993;47(1).

[63] Wallerstein N, Duran B Community- based participatory research contributions to intervention research: the intersection of science and practice to improve health equity. Am J Public Health. 2010;100(4):40. 10.2105/AJPH.2009.184036 PMC2837458 20147663

[64] Smith LT Decolonizing methodologies: research and indigenous peoples. London: Zed Books; 1999 p. 208.

